# Immune Checkpoint Inhibitor-Associated Cardiotoxicity in Solid Tumors: Real-World Incidence, Risk Factors, and Prognostic Analysis

**DOI:** 10.3389/fcvm.2022.882167

**Published:** 2022-05-20

**Authors:** Xue Chen, Aimin Jiang, Rui Zhang, Xiao Fu, Na Liu, Chuchu Shi, Jingjing Wang, Xiaoqiang Zheng, Tao Tian, Xuan Liang, Zhiping Ruan, Yu Yao

**Affiliations:** Department of Medical Oncology, The First Affiliated Hospital of Xi’an Jiaotong University, Xi’an, China

**Keywords:** solid tumor, cardiotoxicity, rechallenge, risk factors, immune checkpoint inhibitor (ICI), prognosis

## Abstract

**Background:**

Immune checkpoint inhibitors (ICIs) have achieved acknowledged progress in cancer therapy. However, ICI-associated cardiotoxicity as one of the most severe adverse events is potentially life-threatening, with limited real-world studies reporting its predictive factors and prognosis. This study aimed to investigate the real-world incidence, risk factors, and prognosis of ICI-related cardiotoxicity in patients with advanced solid tumors.

**Methods:**

Electronic medical records from patients with advanced solid tumors receiving ICIs in the First Affiliated Hospital of Xi’an Jiaotong University were retrospectively reviewed. All patients were divided into the cardiotoxicity group and control group, with logistic regression analysis being implemented to identify potential risk factors of ICI-related cardiotoxicity. Furthermore, survival analysis was also performed to investigate the prognosis of patients with ICI-related cardiotoxicity.

**Results:**

A total of 1,047 participants were enrolled in this retrospective study. The incidence of ICI-related cardiotoxicity in our hospital is 7.0%, while grade 3 and above cardiotoxicity was 2.4%. The logistic regression analysis revealed that diabetes mellitus [odds ratio (OR):1.96, 95% confidence Interval (CI): 1.05–3.65, *p* = 0.034] was an independent risk factor, whereas baseline lymphocyte/monocyte ratio (LMR) (OR: 0.59, 95% CI: 0.36–0.97, *p* = 0.037) was the protective factor of ICI-related cardiotoxicity. Survival analysis indicated that severe cardiotoxicity (≥grade 3) was significantly correlated with bleak overall survival (OS) than mild cardiotoxicity (≤grade 2) (8.3 months vs. not reached, *p* = 0.001). Patients with ICI-related overlap syndrome had poorer overall survival than patients with mere cardiotoxicity (9.4 vs. 24.7 months, *p* = 0.033). However, the occurrence of ICI-related cardiotoxicity was not significantly associated with the OS of overall population with solid tumors. Subgroup analysis showed that lung cancer and PD-L1 usage were significantly correlated with a higher incidence of severe cases.

**Conclusion:**

Immune checkpoint inhibitor-related cardiotoxicity is more common in the real-world setting than the previously published studies. Diabetes mellitus and baseline LMR are the potential predictive biomarkers of ICI-related cardiotoxicity. Although ICI-related cardiotoxicity is not correlated with the prognosis of these patients in our cohort, a systematic and comprehensive baseline examination and evaluation should be performed to avoid its occurrence.

## Introduction

Immune checkpoint inhibitors (ICIs) mainly act on the activation of T cells to fight against tumor cells. There are currently FDA-approved drugs that target cytotoxic T-lymphocyte-associated antigen-4 (CTLA-4), programmed cell death protein 1 (PD-1), and programmed cell death ligand 1 (PD-L1). Although ICI-related adverse effects are overall less severe than chemotherapy, there are potentially lethal adverse effects, such as cardiotoxicity, neuromuscular toxicity, and pulmonary toxicity.

The first case of fatal ICI-related myocarditis was reported by Läubli et al. in 2015 (1), in a 73-year-old patient with metastatic melanoma who developed ICI-associated myocarditis after receiving pembrolizumab, leading to acute heart failure. Two cases of fulminant myocarditis were reported in 2016. Both patients became symptomatic within 2 weeks of administration and aggravated rapidly, leading to death within a short period despite the admission of high-dose glucocorticoid and supportive care. These two cases of severe cardiotoxicity warned clinicians of the security of ICIs (2).

Cardiotoxicity associated with ICIs is broadly classified into myocarditis, pericarditis, and arrhythmia. Other clinical manifestations, such as hypertension, Takotsubo-like syndrome, myocardial ischemia, and myocardial infarction, are less reported (3, 4). In addition, some studies have roughly divided them into inflammatory (including myocarditis, pericarditis, etc.) and non-inflammatory (arrhythmia, myocardial infarction, etc.).

Early studies showed that the incidence of ICI-related cardiotoxicity is less than 1% (5). However, as Ganatra et al. (6) stated, the increasing application of ICIs in real-world and the diverse presentation of cardiotoxicity suggest a higher incidence of cardiotoxicity than previously reported. In addition, subclinical cases and increased awareness of toxicity may also be the contributing factors. Among these classifications, myocarditis maintained the highest incidence and fatality rate, as well as the worst prognosis. However, the presentations of cardiotoxicity in real-world were not separated. It is not rare for myocarditis to occur simultaneously with arrhythmia or pericarditis. Studies have found that approximately 19% of patients with pathologically defined cardiotoxicity developed arrhythmias (7). Two patients with fulminant myocarditis reported in 2016 also developed complete atrioventricular conduction block (2). The pathophysiological manifestation suggested the infiltrated T cells and macrophages in the conduction system. As reported, atrial fibrillation, ventricular arrhythmias, and conduction disturbances were detected in 17–30% of patients with ICI-related cardiotoxicity, of whom 3–13% had mere arrhythmias without myocarditis (8). Although the mechanism is unclear, the investigators concluded that there may be underlying undiagnosed myocarditis (8). There are limited studies on ICI-related pericardial disease. In addition, the incidence is unclear due to the lack of cardiovascular monitoring during ICI clinical trials but maybe more common than previously recognized. In an evaluation based on VigiBase, Salem et al. included 95 cases of pericardial disease associated with ICI, of which 60% were severe and 21% were associated with death (7). Unlike myocarditis, the incidence of pericarditis did not increase significantly with combination immunotherapy. In addition, the study found that more than half of the cases with pericardial diseases were reported in patients with lung cancer (7). Based on the preclinical and clinical studies, several possible influencing factors of ICI-related pericardial involvement were proposed. Given the disproportionately high incidence of pericardial disease in patients with lung cancer and synergy between radiotherapy and immunotherapy is considered a possible driver.

In the previous studies, autoimmune disease, prior heart disease, and combined ICI therapy can be the probable risk factors. Results are mostly derived from databases based on overall irAEs with few real-world studies based on cardiotoxicity. Therefore, we conducted a real-world retrospective study based on the whole immunotherapy population to explore the risk factors for ICI-related cardiotoxicity.

## Materials and Methods

### Patients

Patients diagnosed with advanced solid tumors who were hospitalized from January 2020 to June 2021 at the departments of the medical oncology, surgical oncology, and radiation oncology at the First Affiliated Hospital of Xi’an Jiaotong University were included. We retrospectively screened patients into two groups according to whether cardiotoxicity happened during the administration of ICIs. Suspected cases were assessed mainly according to Bonaca diagnostic criteria (9). There were also cases that does not meet the criterion. In addition to that, we also consider ICI-related myocarditis according to the following two points:

1. Concurrent multi-organ damage with abnormal cardiac biomarkers responding to corticosteroids. Infection and autoimmune diseases were excluded.
2. Dynamical change in cardiac biomarker with ICI medication responding to corticosteroids. Infection and autoimmune diseases were excluded.

Immune checkpoint inhibitor-related pericarditis and arrhythmia were diagnosed according to Naranjo score: Suspected patients who suffered from cardiac damage during ICI therapy with a Naranjo scores ≥ 5 were included in the cardiotoxicity group.

Patients were categorized as “severe” cardiotoxicity if they displayed a grade 3–5 toxicity according to criteria adapted from the Common Terminology Criteria for Adverse Events V.5.0 (Table 1). Patients were categorized as “mild” cardiotoxicity if they displayed a grade 1–2 toxicity.

**TABLE 1:** Criteria for myocarditis severity scoring.

Grade	Criteria
1^a^	Elevated biomarkers without symptoms^b^ (e.g., dyspnea, chest pain, etc.)
2	Elevated biomarkers without symptoms but not requiring patient hospitalization
3	Elevated biomarkers without symptoms requiring patient hospitalization (not requiring intensive care unit level of care); abnormal cardiovascular diagnostic studies (echocardiography showing reduction in LV function or wall motion abnormalities; abnormal cardiac MRI)
4	Deterioration of grade 3 clinical status or requirement for ICU level of care for cardiac symptoms with evidence of decreased cardiac output (cardiogenic shock) or arrhythmia
5	Death of the patient refractory to medical therapy

*LV, left ventricular; MRI, magnetic resonance imaging; ICU, intensive care unit.*

*^a^CTCAE5.0 released in 2017 does not include grade 1 myocarditis. But this criterion cannot reflect the whole situation of the occurrence of myocarditis. This table is based on Bonaca and CTCAE4.0.*

*^b^Biomarkers for myocarditis are markers of myonecrosis, including cardiac troponin, CK-MB, or total CK.*

### Statistical Analysis

Continuous variables are statistically described by means ± standard deviation (subject to normal distribution) or interquartile spacing (not subject to normal distribution); categorical variables are expressed in terms of number and percentage of cases. The SW test was used for the normality test, and one-way ANOVA was used for the variance homogeneity test. If the continuous variable obeys the normal distribution and the variance is homogeneous, the independent sample *t*-test is used, otherwise, the non-parametric rank sum test is used for statistical analysis. Categorical variables were statistically analyzed by the chi-square test or Fisher’s exact probability. All patients were divided into occurrence group and non-occurrence group based on whether cardiotoxicity occurred during ICI treatment, with the univariate logistic regression analysis being exploited to investigate the possible risk factors of cardiotoxicity. Ultimately, the variables with *p* < 0.05 in univariate analysis were included in multivariate logistic regression analysis. All statistical analyses were performed in SPSS 23.0 and R 4.1.1 for Windows 64.0. Multiple imputations were used to supplement missing data.

### Ethical Approval and Informed Consent

This study was conducted in strict accordance with the requirements of the Declaration of Helsinki. Our research also passed the ethics review of the First Affiliated Hospital of Xi’an Jiaotong University (no: XJTU1AF2020LSK-262).

## Results

### Characteristics of the Whole Population

From January 2020 to July 2021, a total of 1,492 patients with advanced solid tumors were hospitalized and treated with ICIs in the departments of medical oncology, surgical oncology, and radiation oncology. A total of 391 patients who did not meet the requirements were excluded, and a total of 1,101 patients who met the research criteria were screened. There were 127 patients with cardiac damage, of which 54 patients did not meet the diagnostic criteria. In the end, 974 patients did not develop cardiotoxicity, and 73 patients developed ICI-related cardiotoxicity, including 25 patients with severe cardiotoxicity and 48 patients with mild cardiotoxicity (Table 2).

**TABLE 2:** Characteristics of the whole population.

Characteristics	Groups	Number of patients (ratio)^a^		
		Without cardiotoxicity (*N* = 974)	With cardiotoxicity (*N* = 73)	*p-*value
Weight		62.2 ± 11.2	63.2 ± 9.4	0.447
Age		59.5 ± 11.2	59.8 ± 11.7	0.835
Gender	Male	657 (67.5%)	56 (76.7%)	0.132
	Female	317 (32.5%)	17 (23.3%)	
Cardiac disease	No	906 (93.0%)	67 (91.8%)	0.872
	Yes	68 (7.0%)	6 (8.2%)	
Hypertension	No	750 (77.0%)	56 (76.7%)	1.000
	Yes	224 (23.0%)	17 (23.3%)	
Diabetes	No	873 (89.6%)	59 (80.8%)	0.033
	Yes	101 (10.4%)	14 (19.2%)	
Cigarette	No	625 (64.2%)	43 (58.9%)	0.437
	Yes	349 (35.8%)	30 (41.1%)	
Family history	No	894 (91.8%)	64 (87.7%)	0.318
	Yes	80 (8.2%)	9 (12.3%)	
Respiratory tumors		329 (33.8%)	31 (42.5%)	0.159
Digestive tumors		467 (47.9%)	29 (39.7%)	0.183
Urinary tract tumors		74 (7.6%)	9 (12.3%)	0.173
Malignant melanoma		39 (4.0%)	2 (2.7%)	1.000
Other tumors^b^		65 (6.7%)	2 (2.7%)	0.315
Prior chemotherapy	No	482 (49.5%)	38 (52.1%)	0.763
	Yes	492 (50.5%)	35 (47.9%)	
Prior antiangiogenic therapy	No	837 (85.9%)	62 (84.9%)	0.950
	Yes	137 (14.1%)	11 (15.1%)	
Treatment line	First-line	486 (49.9%)	39 (53.4%)	0.646
	Post-line	488 (50.1%)	34 (46.6%)	
Lung metastasis	No	750 (77.0%)	60 (82.2%)	0.380
	Yes	224 (23.0%)	13 (17.8%)	
Liver metastasis	No	731 (75.1%)	48 (65.8%)	0.106
	Yes	243 (24.9%)	25 (34.2%)	
Brain metastasis	No	909 (93.3%)	72 (98.6%)	0.121
	Yes	65 (6.7%)	1 (1.4%)	
Bone metastasis	No	764 (78.4%)	60 (82.2%)	0.544
	Yes	210 (21.6%)	13 (17.8%)	
ECOG	0–1	803 (82.4%)	63 (86.3%)	0.496
	2–3	171 (17.6%)	10 (13.7%)	
Treatment	ICI monotherapy	66 (6.8%)	6 (8.2%)	0.818
	ICI combined with chemotherapy	908 (93.2%)	67 (91.8%)	
ICI agent	PD-1	898 (92.2%)	65 (89.0%)	0.463
	PD-L1	76 (7.8%)	8 (11.0%)	
LDH (U/L)	<250	505 (70.0%)	41 (70.7%)	1.000
	≥250	216(30.0%)	17 (29.3%)	
CK (U/L)	<310	701 (98.9%)	56 (98.2%)	1.000
	≥310	8 (1.1%)	1 (1.8%)	
CK-MB (U/L)	<24	599 (84.5%)	45 (78.9%)	0.362
	≥24	110(15.5%)	12 (21.1%)	
Hemoglobin (g/L; normal range 115–150)		126.7 ± 19.9	129.2 ± 18.8	0.296
Platelet count (×10^9^/L; normal range 125–350)		234.3 ± 97.0	214.3 ± 98.5	0.091
White-cell count (×10^9^/L; normal range 4.0–10.0)		6.9 ± 3.3	6.9 ± 2.6	0.892
Neutrophil count (×10^9^/L; normal range 1.8–6.3)		4.9 ± 3.6	4.9 ± 2.5	0.937
Lymphocyte count (×10^9^/L; normal range 1.1–3.2)		1.4 ± 0.6	1.4 ± 0.5	0.203
Monocytes (10^9^/L; normal range 0.1–0.6)		0.4 ± 0.2	0.5 ± 0.4	0.166
Eosinophils (10^9^/L; normal range 0.02–0.52)		0.2 ± 0.5	0.2 ± 0.3	0.482
Albumin (g/L; normal range 40–55)		39.2 ± 4.6	38.7 ± 4.6	0.379
Globulin (g/L; normal range 20–40)		29.5 ± 5.2	29.5 ± 5.0	0.958
TC (mmol/L; 3.10–5.69)		4.3 ± 1.0	4.5 ± 1.3	0.428
PLR		197.3 ± 156.9	183.3 ± 116.8	0.341
NLR		4.2 ± 4.6	4.4 ± 4.2	0.653
LMR		3.9 ± 3.4	3.3 ± 1.7	0.010
A/G		1.4 ± 0.3	1.3 ± 0.3	0.442
Neutrophil ratio (normal range 40–75%)		68.8% ± 39.6%	68.7% ± 10.0%	0.923
Lymphocyte ratio (normal range 20–50%)		23.0% ± 10.2%	21.1% ± 8.3%	0.066

*ECOG, Eastern Cooperative Oncology Group; ICI, immune checkpoint inhibitor; LDH, lactate dehydrogenase; CK, creatine kinase; TC, total cholesterol; PLR, platelet to lymphocyte ratio; NLR, neutrophil to lymphocyte ratio; LMR, lymphocyte to monocyte ratio; A/G, albumin to globulin ratio.*

*^a^Continuous variables are expressed as mean and standard deviation.*

*^b^Others include skin tumors, mediastinal tumors, head and neck tumors, genital neoplasm, sarcomas, and other tumor types.*

Among the 1,047 patients treated with ICI, there were 713 male patients (68.1%) and 334 female patients (31.9%). Most of the patients were younger than 65 years (673 cases, 64.3%), and the physical performance score was mostly 0–1 (866 cases, 82.7%). In terms of cancer types, respiratory tumors (360 cases, 34.4%) and digestive tumors (496 cases, 47.4%) accounted for the majority, followed by urethral tumors (83 cases, 7.9%) and malignant melanoma (43 cases, 3.9%). Other tumor types included skin tumors, mediastinal tumors, head and neck tumors, genital neoplasm, sarcomas, and other tumor types. There were 220 (44.4%) gastrointestinal patients involved in the digestive tumor group, whereas 78 patients (21.7%) with small-cell lung cancer were involved in the respiratory tumor groups. Heart disease (74 cases, 7.1%), diabetes (115 cases, 10.9%), and hypertension (241 cases, 23.0%) were the common comorbidities. There were 379 patients (36.2%) with a history of smoking and 89 patients (8.5%) with a family history of tumors. Most patients received ICI combined chemotherapy (975 cases, 93.1%). PD-1 was the most predominantly used ICIs in our cohort, accounting for 92.0% of cases. In the case of later-line immunotherapy, prior treatment included chemotherapy, radiotherapy, targeted therapy, and antiangiogenic therapy. During the course of immunotherapy, 128 patients developed ICI-associated pneumonia (12.2%), and 380 patients developed ICI-associated thyroid dysfunction (36.3%).

### Risk Factors of Immune Checkpoint Inhibitor-Related Cardiotoxicity

Among the enrolled patients, 73 (7.0%) developed cardiotoxicity, including 48 mild cases (4.6%) and 25 severe cases (2.4%). Among the 115 patients with combined diabetes, 14 developed cardiotoxicity (12.2%). Whereas the occurrence rate of ICI-related cardiotoxicity among patients without diabetes was 6.3%, difference between these two groups was statistically significant (*p* = 0.033). Besides, patients who developed cardiotoxicity have lower LMR (*p* = 0.01). Respiratory tumors maintained the highest incidence of cardiotoxicity among all cancer types (8.6%), but there was no significant difference in the incidence of cardiotoxicity among tumor types. Combined cardiac diseases, ICI agents, history of previous antitumor therapy, and treatment modes have no correlation with the occurrence of cardiotoxicity. In terms of baseline laboratory levels, patients who suffered from cardiotoxicity have a lower lymphocyte ratio than patients without cardiotoxicity. But the difference was not statistically significant (*p* = 0.066). In addition, our research showed that the occurrence of ICI-related pneumonia (*p* = 0.005) and thyroid dysfunction (*p* < 0.005) during the course of ICI treatment were significantly associated with the occurrence of cardiotoxicity. Although most of the other two toxicities appear before cardiotoxicity, we cannot yet explain the relationship between ICI-related cardiotoxicity and ICI-related pneumonia or thyroid dysfunction. But this correlation suggests a link between adverse effects of different systems, and the presence of one toxicity may warn of another that may be more serious.

**FIGURE 1:**
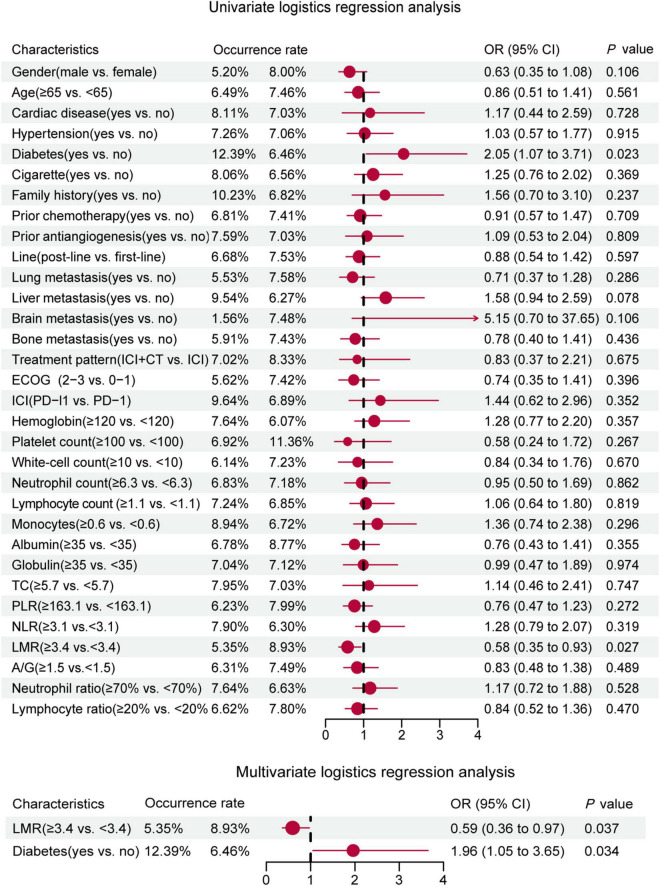
Univariate and multivariate logistic regression forest plot of baseline characteristics and incidence of cardiotoxicity in the whole population.

The univariate logistic regression analysis found that combined diabetes (OR = 2.05, 95% CI: 1.07–3.71, *p* = 0.023) may be a risk factor for cardiotoxicity, whereas lower baseline LMR (OR = 0.58, 95% CI: 0.35–0.93, *p* = 0.027) may be a protective factor (Figure 1). Ultimately, the multivariate regression analysis suggested that diabetes (OR = 1.96, 95% CI: 1.05–3.65, *p* = 0.034) was an independent risk factor for the occurrence of ICI-related cardiotoxicity. In addition, lower baseline LMR (OR = 0.59, 95% CI: 0.36–0.97, *p* = 0.037) was a protective factor (Figure 1).

### Severity of Myocarditis

The population with ICI-related cardiotoxicity included 48 mild cases and 25 severe cases (Table 3). Among them, 4 patients (5%) died in hospital. All in-hospital died patients were severe cardiotoxicity. When cardiotoxicity occurs, echocardiography was abnormal in 20 cases (26.3%), mainly with abnormal wall motion. A total of 24 patients (31.6%) had an abnormal electrocardiogram, including ST-T abnormality, conduction block, and other changes. In addition, 30 patients (41.1%) in the subgroup suffered from cardiotoxicity and concurrent ICI damage of other systems, among which abnormal liver function, myositis, skin rash, and abnormal thyroid function were more common.

**TABLE 3:** Characteristics of patients with cardiotoxicity.

Characteristics	Groups	≤Grade 2 (*N* = 48)	≥Grade3 (*N* = 25)	*p-*value
Age	<65 years old	33 (68.8%)	16 (64%)	0.883
	≥65 years old	15 (31.2%)	9 (36%)	
Gender	Male	38 (79.2%)	18 (72%)	0.692
	Female	10 (20.8%)	7 (28%)	
ECOG	0–1	43 (89.6%)	20 (80%)	0.440
	2–3	5 (10.4%)	5 (20%)	
Cardiac disease	No	44 (91.7%)	23 (92%)	1.000
	Yes	4 (8.3%)	2 (8%)	
Diabetes	No	38 (79.2%)	21 (84%)	0.854
	Yes	10 (20.8%)	4 (16%)	
Respiratory tumors		16 (33.3%)	15 (60%)	0.045
Digestive tumors		24 (50%)	5 (20%)	0.014
Urinary tract tumors		6 (12.5%)	3 (12%)	0.476
Malignant melanoma		1 (2.1%)	1 (4%)	-
Other tumors		1 (2.1%)	1 (4%)	-
Treatment^a^	ICI monotherapy	4 (8.3%)	2 (8%)	1.000
	ICI combined with chemotherapy	44 (91.7%)	23 (92%)	
ICI	PD-1	46 (95.8%)	19 (76%)	0.029
	PD-L1	2 (4.2%)	6 (24%)	
Neutrophil count (×10^9^/L; normal range 1.8–6.3)	<6.3	38 (79.2%)	21 (84%)	0.854
	≥6.3	10 (20.8%)	4 (16%)	
Lymphocyte count (×10^9^/L; normal range 1.1–3.2)	<1.1	14 (29.2%)	9 (36%)	0.741
	≥1.1	34 (70.8%)	16 (64%)	
Monocytes (10^9^/L; normal range 0.1–0.6)	<0.6	35 (72.9%)	22 (88%)	0.238
	≥0.6	13 (27.1%)	3 (12%)	
PLR	<163.1	28 (58.3%)	13 (52%)	0.788
	≥163.1	20 (41.7%)	12 (48%)	
NLR	<3.1	20 (41.7%)	12 (48%)	0.788
	≥3.1	28 (58.3%)	13 (52%)	
LMR	<3.4	31 (64.6%)	14 (56%)	0.644
	≥3.4	17 (35.4%)	11 (44%)	
A/G	<1.5	36 (75%)	16 (64%)	0.476
	≥1.5	12 (25%)	9 (36%)	
Neutrophil ratio (normal range 40–75%)	<70%	22 (45.8%)	14 (56%)	0.563
	≥70%	26 (54.2%)	11 (44%)	
Lymphocyte ratio (normal range 20–50%)	<20%	22 (45.8%)	11 (44%)	1.000
	≥20%	26 (54.2%)	14 (56%)	

*ECOG, Eastern Cooperative Oncology Group; ICI, immune checkpoint inhibitor; PLR, platelet to lymphocyte ratio; NLR, neutrophil to lymphocyte ratio; LMR, lymphocyte to monocyte ratio; A/G, albumin to globulin ratio.*

*^a^Others include prostatic cancer, mediastinal tumor, head and neck squamous cell carcinoma, sarcoma.*

**FIGURE 2:**
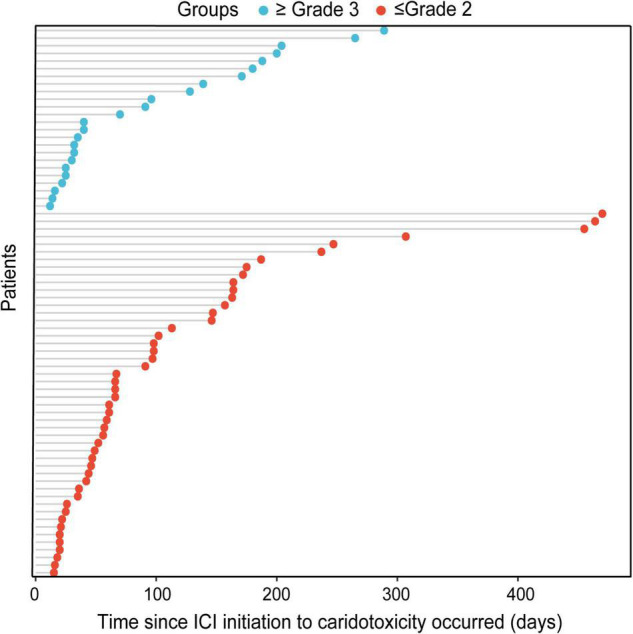
Bubble chart of the time since ICI initiation to the occurrence of mild cardiotoxicity and severe cardiotoxicity.

Cardiotoxicity happened mainly in men (56, 76.7%). In terms of cancer types, the proportion of severe cases in respiratory tumors was significantly higher than in other tumors (*p* = 0.045), whereas the proportion in digestive tumors was significantly lower than other tumors (*p* = 0.014). The ICI agents were mainly PD-1 (65, 89.0%), of which 19 cases (29.2%) were severe, whereas 8 cases received PD-L1, and 6 cases (75.0%) were severe. The difference between the two groups was statistically significant (*p* = 0.008). We did not find that inflammatory parameters, such as NLR, PLR, and LMR, were associated with the severity of cardiotoxicity. Other indicators, including ECOG score, comorbidities, smoking history, family history, prior antitumor therapy, and treatment mode, were not significantly associated with the severity of cardiotoxicity. In addition, unlike the general population, the occurrence of ICI-related thyroid dysfunction (*p* = 0.120) and ICI-related pneumonia (*p* = 0.327) was not significantly associated with the severity of cardiotoxicity. There was no statistically significant difference in the occurrence time of mild and severe cardiotoxicities (median occurrence time: 105 vs. 134 days, *p* = 0.345) (Figure 2).

### Survival Analysis

**FIGURE 3:**
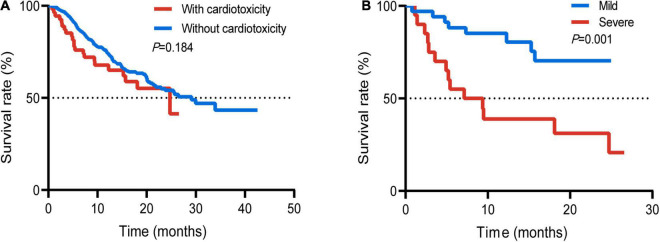
Survival curve of the whole population with or without cardiotoxicity **(A)**; patients with mild or severe cardiotoxicity **(B)**.

Survival analysis was performed in patients with or without cardiotoxicity. We investigated the difference with overall survival in these patients. The median follow-up time was 19.0 months. There was no significant difference with median overall survival time (mOS) between the two groups without cardiotoxicity and those with cardiotoxicity (29.1 vs. 24.7 months, *p* = 0.184) (Figure 3A). The mOS of severe cardiotoxicity was 8.3 months, while the mOS of mild cardiotoxicity was not reached (*p* = 0.001) (Figure 3B).

**FIGURE 4:**
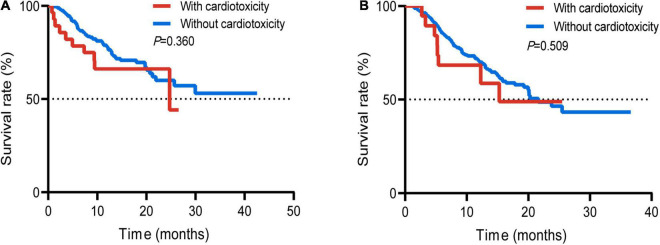
Survival curve of the patients suffered respiratory tumor with or without cardiotoxicity **(A)**; patients suffered digestive tumor with or without cardiotoxicity **(B)**.

Survival analysis was performed on the subgroups of patients with respiratory tumors and digestive tumors due to different prognoses of different tumor types. There was no statistically significant difference between the two groups with or without cardiotoxicity in the respiratory tumors (*p* = 0.360) (Figure 4A). The mOS between the two groups without cardiotoxicity in the digestive system and those with cardiotoxicity was 21.6 and 15.3 months, respectively (Figure 4B). But the difference was also not statistically significant (*p* = 0.509). For all patients with cardiotoxicity, mOS was 17.0 months.

**FIGURE 5:**
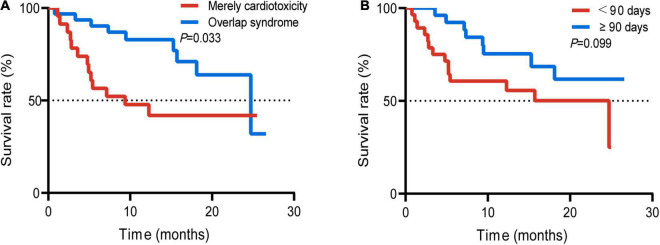
Survival curve of the patients with mere cardiotoxicity and patients with concurrent cardiotoxicity and ICI targeted other functional disorder **(A)**; occurrence time (time since ICI initiation to the occurrence of ICI-related cardiotoxicity) of 90 days before and after 90 days **(B)**.

In addition, we also compared the survival difference between the groups with mere cardiotoxicity and those with concurrent cardiotoxicity and other toxicities. The mOS with mere cardiotoxicity was 24.7 months, and the mOS in the group with overlap syndrome was 9.4 months (Figure 5A). The difference was statistically significant (*p* = 0.033). In 2020, Dolladille et al. (10) analyzed the clinical characteristics of early and late cardiac adverse reactions through retrospective analysis of multicenter cases and data from the VigiBase using 90 days as a cutoff. They found differences in the characteristics of early and late cardiac cardiotoxicities. We furtherly plotted the survival curves of the two groups of patients with the occurrence time before 90 days and after 90 days. However, there was no significant difference between the two groups (Figure 5B).

### Immune Checkpoint Inhibitor Rechallenge

**FIGURE 6:**
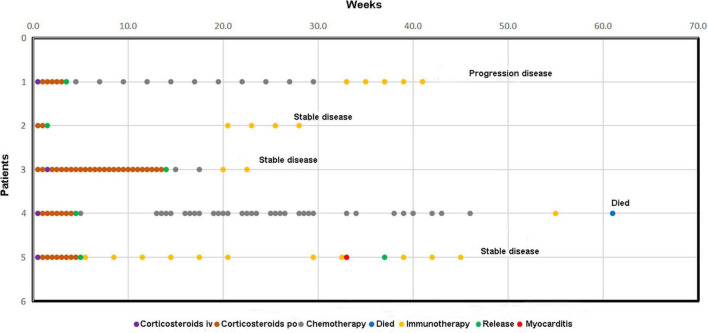
Follow-up of patients who restart ICI therapy after suffering ICI-related cardiotoxicity.

Current guidelines suggest that restarting ICI is not recommended for grade 2 and above cardiotoxicity. We also strictly follow the recommendations in our clinical work. However, the complexity of real-world research results in patient diversity. In our study, some patients persisted in restarting ICI despite being fully informed of the risks. In the follow-up of patients who developed cardiotoxicity, there were 5 additional patients with grades 2 and 3 myocarditis who readmitted ICI after recovery (Figure 6). Only one of the five patients who had grade 3 myocarditis suffered grade 2 myocarditis after the rechallenge of ICI. No other grade 3 and above ICI toxicity were found in all patients. Following up for more than 1 year, only one case died of disease progression. The remaining patients were all alive. According to our investigation, the ICI rechallenge seems relatively secure. However, considering the small sample size in our cohort, more relevant studies are needed in the future to identify the safety of ICI rechallenge.

## Discussion

We found that the incidence of cardiotoxicity was 7.0%, which was higher than the previously reported. By collecting the cardiovascular risk factors of the enrolled patients, we found that diabetes was an independent risk factor for the occurrence of cardiotoxicity. This conclusion can be seen in the retrospective study of Mahmood et al. (11). Through a multicenter retrospective study of 35 patients, they found that myocarditis was more common in patients with diabetes mellitus, especially in patients receiving a combination of ICI therapy. Although they ultimately failed to prove it an independent risk factor, their research suggested a certain direction. In our analysis, an exact association of diabetes with the development of cardiotoxicity was found. However, we did not find an association between other cardiovascular risk factors, such as hypertension and the occurrence of cardiotoxicity.

The effect of diabetes on the development of ICI-related cardiotoxicity may be related to the long-term chronic inflammation in patients with diabetes. Till now, diabetes has been regarded as a chronic inflammatory disease. The high-glucose environment of diabetes significantly increases the cytokines, such as interleukin 4(IL-4), interleukin 5(IL-5), interleukin 6(IL-6), interleukin 13(IL-13), and tumor necrosis factorα (TNF-α). These cytokines maintained the balance of the autoimmune microenvironment. The chronic pathological state of immune imbalance caused by diabetes can promote many diseases (12–14). In addition, the oxidative stress produced by diabetes can also produce many inflammatory cytokines, such as TNF-α, IL-6, and transforming growth factor β (14). The cytokine pathway has been confirmed to be closely related to irAEs in many studies (15). In addition, diabetes affects the number and activity of T cells, B cells, and NK cells in peripheral blood, and the result of immune imbalance may also play an important role (14). Moreover, whether diabetes, as an autoimmune disease, also plays an important role in irAEs like other types of autoimmune diseases remains to be further studied.

We also collected baseline inflammatory indicators, such as IL-6, C-reactive protein (CRP), and lactic dehydrogenase (LDH), of all patients and calculated NLR, PLR, and LMR. We found that lower baseline LMR was an independent risk factor for the development of cardiotoxicity. In previous studies, the correlation between peripheral lymphocyte levels and adverse reactions has already been investigated. In a study of risk factors for cardiotoxicity based on more than 4,000 immunotherapy patients (16), the authors identified the association between low baseline peripheral lymphocyte levels and cardiotoxicity through a machine learning method. In a retrospective study of NSCLC, an association of LMR with irAEs was also found (17). Activated T lymphocytes in patients with tumors after using PD-1/PD-L1 antibodies can not only attack tumor cells, but also cause irAEs. According to the autopsy result of patients with cardiotoxicity as previously reported, lymphocytic infiltration was widely seen in the diseased tissue (2). Therefore, lymphocytes may play a key role in the response of irAEs. However, no direct correlation between peripheral lymphocyte count (or percentage) and the occurrence of cardiotoxicity was found in our cohort. Moreover, these indicators are often dynamic during tumor treatment. Therefore, how does their changing act on adverse reactions? What role do lymphocytes play in it? They deserve further discussion.

In terms of gender, ICI was used in more men than women in our study, possibly due to the higher tumor prevalence in male patients. In this study, male patients received ICI proportionally more often than female patients, but no significant association between gender and the development of myocarditis was found. One study found higher occurrence rate of ICI associated cardiovascular with men (18). Several additional studies had the same finding (7, 11, 19). However, another research found a different result that women were at higher risk of ICI-related myocarditis. These studies suggested the relationship of gender and the occurrence of ICI-related myocarditis. In addition, we found a higher incidence of ICI-related cardiotoxicity in patients with respiratory neoplasms than in other types of neoplasms. Salem et al. (7) also found a higher rate of ICI-related pericardial disease among patients with lung cancer. Basic research has found upregulation of PD-L1 in models of myocardial injury induced by ischemiareperfusion and hypothermia, which may be a cytokine-mediated mechanism of cardiac protection. Besides, radiation therapy in patients with lung cancer may present with potential exposure to cardiac antigens. Immune activation thereby conferring them a higher incidence of cardiotoxicity. As for the correlation between comorbid autoimmune diseases and cardiotoxicity, there were numerous reports of thymoma patients with ICI-related cardiotoxicity. Toi et al. (20) retrospectively recruited 137 patients with positive autoimmune antibody receiving ICI therapy. They found that positive autoimmune antibody was significantly associated with a higher occurrence of irAEs and better clinical benefit in NSCLC. In addition, another study also suggested a relationship between autoimmune antibodies and irAEs (20). Therefore, we are more cautious about ICI initiation in patients with autoimmune diseases in real-world. Therefore, patients with active autoimmune disease were excluded from our study. Because the treatment of active autoimmune diseases is contrary to tumor treatment, they can affect the judgment to response of tumor treatment and drug-related adverse reactions.

In addition to ICI-related cardiotoxicity, we also collected ICI-related thyroid dysfunction and pneumonia. We found that both of them have a certain correlation with the occurrence of cardiotoxicity. Although the onset of ICI-related pneumonitis or abnormal thyroid function does not completely precede the onset of cardiotoxicity, we cannot establish a causal relationship between them. However, this finding suggests that there may be a link between the toxicities occurring at different times targeting different organs. The occurrence of toxicities targeting the lung or thyroid may then alert clinicians the occurrence of cardiotoxicity. Besides, myositis, hepatitis, and pneumonia may be complicated by the occurrence of cardiotoxicity. In the cardiotoxicity population, a total of 30 (41.1%) patients had concurrent toxicities targeting other organs. In a study based on the VigiBase database, serious combined ICI-related adverse reactions accounted for 42% of adverse reactions, most of which were ICI-related myocarditis and myositis (21). In a multicenter study, 32% of myositis was associated with concurrent myocarditis (22). In addition, the mortality rate is the highest in patients with combined myocarditis and myasthenia gravis. Therefore, in patients with symptoms of ICI-related myositis, they should be alert to the occurrence of myocarditis.

Previous studies have demonstrated a possible correlation between irAEs and tumor prognosis. A meta-analysis (23) explored the relationship between ICI-related adverse drug reactions and clinical benefits. It was suggested that in patients receiving ICIs, the development of irAEs was positively correlated with objective remission rate (ORR), progression-free survival (PFS), and OS, irrespective of disease site, type of ICI, and irAE. ORR was better in patients with grade 3 or higher toxicity, but OS was worse. However, in this study, we only did the analysis in overall survival, and no significant difference was found between patients with or without cardiotoxicity.

Till now, the National Comprehensive Cancer Network (NCCN) guidelines do not recommend rechallenge of ICI after grade 2 and above myocarditis. Subclinical myocarditis was recommended continue the use of ICI with close detection. In a single-center retrospective study in 2019 (24), the investigators included 93 patients with grade 2 or higher toxicity, including 43 grade 2 events, 36 grade 3 events, and 14 grade 4 events. Taking the occurrence of the second toxicity as the endpoint, the final results suggested that the time since ICI use to the occurrence of the initial irAE was related to the occurrence of the second irAE. Besides, the severity of the second irAE was not more severe than the first one. They concluded that the risk-reward ratio of anti-PD-1 or anti-PD-L1 rechallenge appears to be acceptable. However, patients with first ICI-related toxicity involving the heart were not included in this study. Conversely, there were also some studies restarting ICI after the occurrence of irAEs, resulting in recurrence of grade 5 toxicity in patients. Therefore, ICI rechallenge after irAEs is still controversial. Due to the small number of cases in our study, we cannot currently explain the safety of cardiotoxicity after restarting, because once cardiotoxicity occurs, it may be fatal drug toxicity, which is unacceptable for clinicians and patients’ families. Moreover, the timing of ICI restarting among these patients also varied, and some patients even resumed ICI 1 month after ICI withdrawal. If there are large clinical studies to confirm the feasibility of restarting immunotherapy for ICI-related cardiotoxicity, the timing of restarting ICI will also be a major focus. Furthermore, there were many patients with grade 1 cardiotoxicity who continue to use immunotherapy under strict monitoring, and a small number of patients who suffered mild cardiotoxicity for not one time, but it does not affect the progress of tumor treatment.

## Conclusion

We retrospectively analyzed the risk factors of ICI-related cardiotoxicity in the whole population receiving ICI therapy. We found a higher incidence of ICI-related cardiotoxicity, and a high proportion of severe cases than previous reported in real-world situation. Patients with diabetes mellitus and low baseline levels of LMR have an increased incidence of cardiotoxicity, which should be closely monitored during the use of ICIs. Besides, the incidence of severe cardiotoxicity was correlated with shorter overall survival.

## Shortage

Despite the advantages and potential insights on this study, there are some inevitable shortcomings in our study. First of all, some potential biases are still unavoidable due to the retrospective design of this study, such as investigator bias. Second, although the overall immunotherapy sample size we included is relatively large, the number of cardiotoxicity cases is relatively small, as well as the number of patients who rechallenge ICI. Finally, due to the serious lack of some data during the study period, we could not analyze the relationship between the baseline levels of some inflammatory markers (such as LDH, CRP, and cytokines) and the occurrence of cardiotoxicity. Hence, well-designed large-scale prospective studies are urgently needed in the future.

## Data Availability Statement

The raw data supporting the conclusions of this article will be made available by the authors, without undue reservation.

## Ethics Statement

This study involving human participants was reviewed and approved by the Ethics Review of the First Affiliated Hospital of Xi’an Jiaotong University (no. XJTU1AF2020LSK-262) and was conducted in strict accordance with the requirements of the Declaration of Helsinki. Written informed consent was not required due to the retrospective nature of the study.

## Author Contributions

XC, TT, XL, ZR, and YY contributed to the design of the study. XC, AJ, RZ, XF, NL, JW, and XZ contributed to manuscript preparation. XC and AJ wrote the manuscript. RZ, YY, CS, and XL helped collect cases. All authors contributed to the article and approved the submitted version.

## Conflict of Interest

The authors declare that the research was conducted in the absence of any commercial or financial relationships that could be construed as a potential conflict of interest.

## Publisher’s Note

All claims expressed in this article are solely those of the authors and do not necessarily represent those of their affiliated organizations, or those of the publisher, the editors and the reviewers. Any product that may be evaluated in this article, or claim that may be made by its manufacturer, is not guaranteed or endorsed by the publisher.

## References

[1] Läubli H Balmelli C Bossard M Pfister O Glatz K Zippelius A. Acute heart failure due to autoimmune myocarditis under pembrolizumab treatment for metastatic melanoma. *J Immunother Cancer.* (2015) 3:11. 10.1186/s40425-015-0057-1 25901283 PMC4404586

[2] Johnson DB Balko JM Compton ML Chalkias S Gorham J Xu Y Fulminant myocarditis with combination immune checkpoint blockade. *N Engl J Med.* (2016) 375:1749–55. 10.1056/NEJMoa1609214 27806233 PMC5247797

[3] Geisler BP Raad RA Esaian D Sharon E Schwartz DR. Apical Ballooning and Cardiomyopathy in a melanoma patient treated with ipilimumab: a case of takotsubo-like syndrome. *J Immunother Cancer.* (2015) 3:4. 10.1186/s40425-015-0048-2 25705383 PMC4335413

[4] Nykl R Fischer O Vykoupil K Taborsky M. A unique reason for coronary spasm causing temporary st elevation myocardial infarction (inferior stemi) - systemic inflammatory response syndrome after use of pembrolizumab. *Arch Med Sci Atheroscler Dis.* (2017) 2:e100–2. 10.5114/amsad.2017.72531 29379889 PMC5777475

[5] Upadhrasta S Elias H Patel K Zheng L. Managing cardiotoxicity associated with immune checkpoint inhibitors. *Chronic Dis Transl Med.* (2019) 5:6–14. 10.1016/j.cdtm.2019.02.004 30993259 PMC6450824

[6] Ganatra S Neilan TG. Immune checkpoint inhibitor-associated myocarditis. *Oncologist.* (2018) 23:879–86. 10.1634/theoncologist.2018-0130 29802219 PMC6156176

[7] Salem JE Manouchehri A Moey M Lebrun-Vignes B Bastarache L Pariente A Cardiovascular toxicities associated with immune checkpoint inhibitors: an observational, retrospective, pharmacovigilance study. *Lancet Oncol.* (2018) 19:1579–89. 10.1016/s1470-2045(18)30608-9 30442497 PMC6287923

[8] Escudier M Cautela J Malissen N Ancedy Y Orabona M Pinto J Clinical features, management, and outcomes of immune checkpoint inhibitor-related cardiotoxicity. *Circulation.* (2017) 136:2085–7. 10.1161/circulationaha.117.030571 29158217

[9] Bonaca MP Olenchock BA Salem JE Wiviott SD Ederhy S Cohen A Myocarditis in the setting of cancer therapeutics: proposed case definitions for emerging clinical syndromes in cardio-oncology. *Circulation.* (2019) 140:80–91. 10.1161/circulationaha.118.034497 31390169 PMC6779326

[10] Dolladille C Ederhy S Allouche S Dupas Q Gervais R Madelaine J Late cardiac adverse events in patients with cancer treated with immune checkpoint inhibitors. *J Immunother Cancer.* (2020) 8:e000261. 10.1136/jitc-2019-000261 31988143 PMC7057417

[11] Mahmood SS Fradley MG Cohen JV Nohria A Reynolds KL Heinzerling LM Myocarditis in patients treated with immune checkpoint inhibitors. *J Am Coll Cardiol.* (2018) 71:1755–64. 10.1016/j.jacc.2018.02.037 29567210 PMC6196725

[12] Parikh RB Min EJ Wileyto EP Riaz F Gross CP Cohen RB Uptake and survival outcomes following immune checkpoint inhibitor therapy among trial-ineligible patients with advanced solid cancers. *JAMA Oncol.* (2021) 7:1843–50. 10.1001/jamaoncol.2021.4971 34734979 PMC8569600

[13] Tanaka S Isoda F Ishihara Y Kimura M Yamakawa T. T Lymphopaenia in relation to body mass index and TNF-alpha in human obesity: adequate weight reduction can be corrective. *Clin Endocrinol (Oxf).* (2001) 54:347–54. 10.1046/j.1365-2265.2001.1139cn2155.x 11298087

[14] Dong G Li Y Zhao Q Pang B Qi X Wei J Effects of diabetes on the development of radiation pneumonitis. *Respir Res.* (2021) 22:160. 10.1186/s12931-021-01754-4 34030688 PMC8147083

[15] Kang JH Bluestone JA Young A. Predicting and preventing immune checkpoint inhibitor toxicity: targeting cytokines. *Trends Immunol.* (2021) 42:293–311. 10.1016/j.it.2021.02.006 33714688

[16] Heilbroner SP Few R Mueller J Chalwa J Charest F Suryadevara S Predicting cardiac adverse events in patients receiving immune checkpoint inhibitors: a machine learning approach. *J Immunother Cancer.* (2021) 9:e002545. 10.1136/jitc-2021-002545 34607896 PMC8491414

[17] Egami S Kawazoe H Hashimoto H Uozumi R Arami T Sakiyama N Peripheral blood biomarkers predict immune-related adverse events in non-small cell lung cancer patients treated with pembrolizumab: a multicenter retrospective study. *J Cancer.* (2021) 12:2105–12. 10.7150/jca.53242 33754009 PMC7974524

[18] Wang DY Salem JE Cohen JV Chandra S Menzer C Ye F Fatal toxic effects associated with immune checkpoint inhibitors: a systematic review and meta-analysis. *JAMA Oncol.* (2018) 4:1721–8. 10.1001/jamaoncol.2018.3923 30242316 PMC6440712

[19] Reuben A Petaccia de Macedo M McQuade J Joon A Ren Z Calderone T Comparative immunologic characterization of autoimmune giant cell myocarditis with ipilimumab. *Oncoimmunology.* (2017) 6:e1361097. 10.1080/2162402x.2017.1361097 29209563 PMC5706622

[20] Toi Y Sugawara S Sugisaka J Ono H Kawashima Y Aiba T Profiling preexisting antibodies in patients treated with Anti-Pd-1 therapy for advanced non-small cell lung cancer. *JAMA Oncol.* (2019) 5:376–83. 10.1001/jamaoncol.2018.5860 30589930 PMC6439838

[21] Moslehi JJ Salem JE Sosman JA Lebrun-Vignes B Johnson DB. Increased reporting of fatal immune checkpoint inhibitor-associated myocarditis. *Lancet.* (2018) 391:933. 10.1016/s0140-6736(18)30533-6 PMC6668330 29536852

[22] Moreira A Loquai C Pföhler C Kähler KC Knauss S Heppt MV Myositis and neuromuscular side-effects induced by immune checkpoint inhibitors. *Eur J Cancer.* (2019) 106:12–23. 10.1016/j.ejca.2018.09.033 30453170

[23] Hussaini S Chehade R Boldt RG Raphael J Blanchette P Maleki Vareki S Association between immune-related side effects and efficacy and benefit of immune checkpoint inhibitors – a systematic review and meta-analysis. *Cancer Treat Rev.* (2021) 92:102134. 10.1016/j.ctrv.2020.102134 33302134

[24] Simonaggio A Michot JM Voisin AL Le Pavec J Collins M Lallart A Evaluation of readministration of immune checkpoint inhibitors after immune-related adverse events in patients with cancer. *JAMA Oncol.* (2019) 5:1310–7. 10.1001/jamaoncol.2019.1022 31169866 PMC6555478

